# *N*,*N*-Dimethyl-3-phenyl­isoxazole-5-carbox­amide

**DOI:** 10.1107/S1600536813033199

**Published:** 2013-12-24

**Authors:** Li Wang, Ya-Jun Li, Zheng-Wei Li, Kai-Ge Liu, Shao-Hua Zhang

**Affiliations:** a Affiliated Hospital of Xi’an Medical College, 48 Fenghao West Road, 710077 Xi’an, People’s Republic of China

## Abstract

In the title compound, C_12_H_12_N_2_O_2_, synthesized by ammonolysis of 3-phenyl­isoxazole-5-carbonyl chloride in di­chloro­methane, the dihedral angle between the isoxazole ring and the phenyl ring is 14.05 (7)°. In the crystal, centrosym­metrically related mol­ecules are linked into dimers by pairs of C—H⋯O hydrogen bonds, generating rings of graph-set motif *R*_2_^2^(10).

## Related literature   

For the biological activity of isoxazole derivatives, see: Lopes *et al.* (2011 ▶). For the synthesis and structure of a related compound, see: Wang *et al.* (2013 ▶).
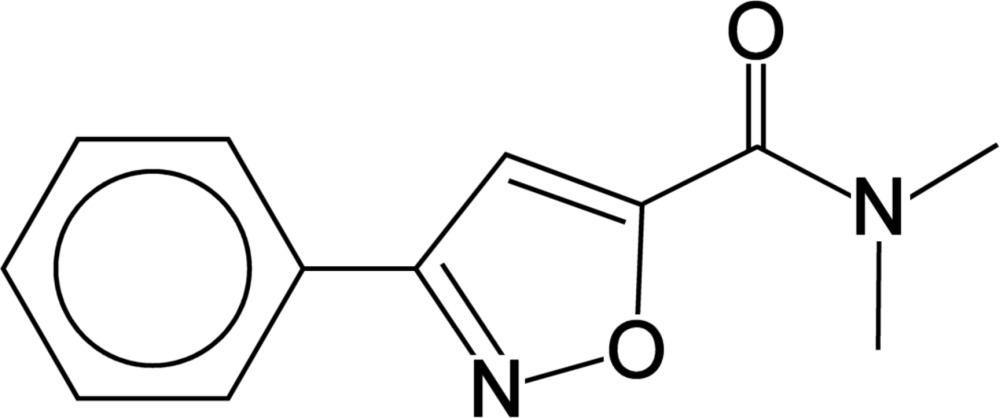


## Experimental   

### 

#### Crystal data  

C_12_H_12_N_2_O_2_
*M* *_r_* = 216.24
Monoclinic, 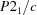

*a* = 7.596 (3) Å
*b* = 12.377 (6) Å
*c* = 12.123 (6) Å
β = 102.964 (8)°
*V* = 1110.7 (9) Å^3^
*Z* = 4
Mo *K*α radiation
μ = 0.09 mm^−1^
*T* = 296 K
0.36 × 0.25 × 0.13 mm

#### Data collection  

Bruker APEXII CCD area detector diffractometer
Absorption correction: multi-scan (*SADABS*; Bruker, 2008 ▶) *T*_min_ = 0.970, *T*_max_ = 0.989
5434 measured reflections
1977 independent reflections
1373 reflections with *I* > 2σ(*I*)
*R*_int_ = 0.032

#### Refinement  

*R*[*F* ^2^ > 2σ(*F* ^2^)] = 0.047
*wR*(*F* ^2^) = 0.128
*S* = 1.03
1977 reflections
148 parameters
H-atom parameters constrained
Δρ_max_ = 0.14 e Å^−3^
Δρ_min_ = −0.17 e Å^−3^

### 

Data collection: *APEX2* (Bruker, 2008 ▶); cell refinement: *SAINT* (Bruker, 2008 ▶); data reduction: *SAINT*; program(s) used to solve structure: *SHELXS97* (Sheldrick, 2008 ▶); program(s) used to refine structure: *SHELXL97* (Sheldrick, 2008 ▶); molecular graphics: *SHELXTL* (Sheldrick, 2008 ▶); software used to prepare material for publication: *publCIF* (Westrip, 2010 ▶).

## Supplementary Material

Crystal structure: contains datablock(s) I. DOI: 10.1107/S1600536813033199/rz5100sup1.cif

Structure factors: contains datablock(s) I. DOI: 10.1107/S1600536813033199/rz5100Isup2.hkl

Supporting information file. DOI: 10.1107/S1600536813033199/rz5100Isup3.cml

Additional supporting information:  crystallographic information; 3D view; checkCIF report

## Figures and Tables

**Table 1:** Hydrogen-bond geometry (Å, °)

*D*—H⋯*A*	*D*—H	H⋯*A*	*D*⋯*A*	*D*—H⋯*A*
C8—H8⋯O2^i^	0.93	2.43	3.340 (3)	165

Symmetry code: (i) 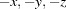.

**Fig. 1.**
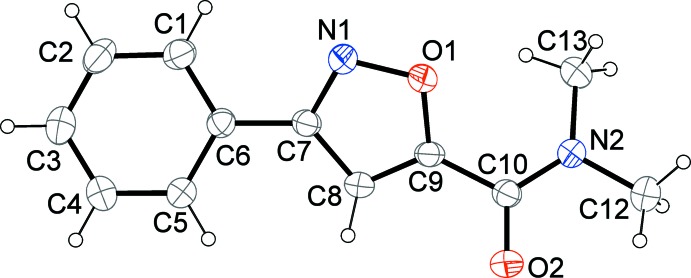
The molecular structure of the title compound with displacement ellipsoids drawn at the 30% probability level.

## supplementary crystallographic information

### 1. Comment

Isoxazoles are reactants or intermediates in the synthesis of compounds that have attracted chemists, biologists and pharmacologists interest. Isoxazole derivatives have been used as semiconductors and corrosion inhibitors. They also show widespread biological activities, and are employed as anthelmintics, antiparasitic, herbicidal, pesticides, fungicide and antiviral drugs (Lopes *et al.*, 2011).

In the molecule of the title compound (Fig. 1), the dihedral angle between the phenyl and the isoxazole rings is 14.05 (7)°, which is bigger than that of 7.37 (19)° observed in the related compound isopropyl 3-phenylisoxazole-5-carboxylate (Wang *et al.*, 2013). The bond lengths within the isoxazole ring are in agreement with those reported for isopropyl 3-phenylisoxazole-5-carboxylate. In the crystal, centrosymmetrically related molecules are linked into dimers by C—H···O hydrogen bonds (Table 1), generating a ring of graph-set motif *R*^2^_2_(10).

### 2. Experimental

3-Phenylisoxazole-5-carboxylic acid (10 mmol, 1.95 g; Wang *et al.*, 2013) was dissolved in 100 ml dichloromethane, then thionyl chloride (12 mmol, 1.43 g) was dropped into the solution and stirred for 20 minutes in ice bath. The solvent was removed under reduced pressure and the mixture was used for the next step without further purification. Dimethylamine (20 mmol, 0.9 g) was added subsequently and the mixture stirred for 6 h at room temperature. The resulting residue was purified as a white solid (1.82 g, 84% yield). Recrystallization in dichloromethane gave fine colourless crystals suitable for X-ray study. All chemicals were purchased by Sigma Aldrich Germany.

### 3. Refinement

All H atoms were placed in idealized positions and allowed to ride on the respective parent atom, with C—H = 0.93–0.96 Å and with *U*_iso_(H) = 1.2 *U*_eq_(C) or 1.5*U*_eq_(C) for methyl H atoms.

### Crystal data

**Table:**

C_12_H_12_N_2_O_2_	*Z* = 4
*M**_r_* = 216.24	*F*(000) = 456
Monoclinic, *P*2_1_/*c*	*D*_x_ = 1.287 Mg m^−^^3^
Hall symbol: -P 2ybc	Mo *K*α radiation, λ = 0.71073 Å
*a* = 7.596 (3) Å	θ = 2.4–25.1°
*b* = 12.377 (6) Å	µ = 0.09 mm^−^^1^
*c* = 12.123 (6) Å	*T* = 296 K
β = 102.964 (8)°	Block, colourless
*V* = 1110.7 (9) Å^3^	0.36 × 0.25 × 0.13 mm

### Data collection

**Table:**

Bruker APEXII CCD area detector diffractometer	1977 independent reflections
Radiation source: fine-focus sealed tube	1373 reflections with *I* > 2σ(*I*)
Graphite monochromator	*R*_int_ = 0.032
phi and ω scans	θ_max_ = 25.1°, θ_min_ = 2.4°
Absorption correction: multi-scan (*SADABS*; Bruker, 2008)	*h* = −5→9
*T*_min_ = 0.970, *T*_max_ = 0.989	*k* = −14→14
5434 measured reflections	*l* = −14→14

### Refinement

**Table:**

Refinement on *F*^2^	Secondary atom site location: difference Fourier map
Least-squares matrix: full	Hydrogen site location: inferred from neighbouring sites
*R*[*F*^2^ > 2σ(*F*^2^)] = 0.047	H-atom parameters constrained
*wR*(*F*^2^) = 0.128	*w* = 1/[σ^2^(*F*_o_^2^) + (0.0624*P*)^2^ + 0.0693*P*] where *P* = (*F*_o_^2^ + 2*F*_c_^2^)/3
*S* = 1.03	(Δ/σ)_max_ < 0.001
1977 reflections	Δρ_max_ = 0.14 e Å^−^^3^
148 parameters	Δρ_min_ = −0.17 e Å^−^^3^
0 restraints	Extinction correction: *SHELXL97* (Sheldrick, 2008), Fc^*^=kFc[1+0.001xFc^2^λ^3^/sin(2θ)]^-1/4^
Primary atom site location: structure-invariant direct methods	Extinction coefficient: 0.007 (2)

### Special details

**Table:**

Geometry. All e.s.d.'s (except the e.s.d. in the dihedral angle between two l.s. planes) are estimated using the full covariance matrix. The cell e.s.d.'s are taken into account individually in the estimation of e.s.d.'s in distances, angles and torsion angles; correlations between e.s.d.'s in cell parameters are only used when they are defined by crystal symmetry. An approximate (isotropic) treatment of cell e.s.d.'s is used for estimating e.s.d.'s involving l.s. planes.
Refinement. Refinement of *F*^2^ against ALL reflections. The weighted *R*-factor *wR* and goodness of fit *S* are based on *F*^2^, conventional *R*-factors *R* are based on *F*, with *F* set to zero for negative *F*^2^. The threshold expression of *F*^2^ > σ(*F*^2^) is used only for calculating *R*-factors(gt) *etc*. and is not relevant to the choice of reflections for refinement. *R*-factors based on *F*^2^ are statistically about twice as large as those based on *F*, and *R*-factors based on ALL data will be even larger.

### Fractional atomic coordinates and isotropic or equivalent isotropic displacement parameters (Å^2^)

**Table:**

	*x*	*y*	*z*	*U*_iso_*/*U*_eq_	
N1	0.4029 (3)	0.01587 (14)	0.33843 (15)	0.0628 (6)	
O1	0.3706 (2)	0.11427 (10)	0.27668 (12)	0.0616 (5)	
O2	0.1335 (2)	0.15233 (11)	−0.00766 (12)	0.0643 (5)	
C1	0.3700 (3)	−0.19723 (17)	0.43043 (18)	0.0575 (6)	
H1	0.4227	−0.1441	0.4815	0.069*	
C2	0.3675 (3)	−0.30437 (19)	0.4660 (2)	0.0659 (7)	
H2	0.4206	−0.3224	0.5405	0.079*	
C3	0.2870 (3)	−0.38412 (18)	0.3915 (2)	0.0654 (7)	
H3	0.2847	−0.4552	0.4160	0.078*	
C4	0.2103 (3)	−0.35728 (18)	0.2807 (2)	0.0654 (7)	
H4	0.1563	−0.4108	0.2305	0.079*	
C5	0.2126 (3)	−0.25108 (17)	0.24309 (18)	0.0538 (6)	
H5	0.1606	−0.2341	0.1681	0.065*	
C6	0.2930 (3)	−0.16985 (15)	0.31781 (16)	0.0448 (5)	
C7	0.2953 (3)	−0.05662 (15)	0.27653 (16)	0.0431 (5)	
C8	0.1948 (3)	−0.00969 (15)	0.17427 (16)	0.0470 (5)	
H8	0.1123	−0.0440	0.1166	0.056*	
C9	0.2449 (3)	0.09524 (15)	0.17905 (16)	0.0440 (5)	
C10	0.1868 (3)	0.18267 (15)	0.09211 (17)	0.0460 (5)	
N2	0.1934 (2)	0.28752 (12)	0.12311 (13)	0.0493 (5)	
C12	0.1405 (4)	0.36960 (17)	0.03430 (19)	0.0701 (7)	
H12A	0.1469	0.3393	−0.0376	0.105*	
H12B	0.2209	0.4303	0.0506	0.105*	
H12C	0.0192	0.3929	0.0319	0.105*	
C13	0.2324 (3)	0.32936 (17)	0.23967 (17)	0.0613 (6)	
H13A	0.2315	0.2708	0.2915	0.092*	
H13B	0.1421	0.3814	0.2472	0.092*	
H13C	0.3491	0.3632	0.2564	0.092*	

### Atomic displacement parameters (Å^2^)

**Table:**

	*U*^11^	*U*^22^	*U*^33^	*U*^12^	*U*^13^	*U*^23^
N1	0.0738 (13)	0.0460 (10)	0.0568 (12)	−0.0028 (9)	−0.0101 (10)	0.0042 (8)
O1	0.0719 (10)	0.0442 (8)	0.0564 (9)	−0.0073 (7)	−0.0118 (8)	−0.0004 (7)
O2	0.0872 (12)	0.0580 (9)	0.0411 (9)	−0.0080 (8)	0.0000 (8)	−0.0026 (7)
C1	0.0584 (14)	0.0616 (13)	0.0488 (13)	−0.0027 (11)	0.0040 (11)	0.0037 (11)
C2	0.0668 (15)	0.0713 (16)	0.0552 (14)	0.0036 (12)	0.0048 (12)	0.0208 (12)
C3	0.0724 (16)	0.0536 (14)	0.0705 (17)	0.0020 (12)	0.0169 (13)	0.0148 (12)
C4	0.0806 (17)	0.0477 (13)	0.0657 (16)	−0.0039 (11)	0.0117 (13)	−0.0004 (11)
C5	0.0626 (14)	0.0483 (12)	0.0475 (12)	0.0016 (10)	0.0060 (11)	0.0026 (10)
C6	0.0430 (11)	0.0442 (11)	0.0458 (11)	0.0019 (9)	0.0073 (9)	0.0020 (9)
C7	0.0422 (11)	0.0431 (11)	0.0421 (12)	0.0000 (9)	0.0052 (9)	−0.0049 (9)
C8	0.0517 (12)	0.0461 (12)	0.0394 (11)	−0.0049 (9)	0.0017 (10)	−0.0041 (9)
C9	0.0457 (11)	0.0465 (11)	0.0368 (11)	−0.0007 (9)	0.0031 (9)	−0.0055 (9)
C10	0.0497 (12)	0.0449 (11)	0.0425 (12)	−0.0056 (9)	0.0087 (10)	−0.0027 (9)
N2	0.0597 (11)	0.0432 (9)	0.0425 (10)	−0.0016 (8)	0.0060 (8)	0.0020 (8)
C12	0.0889 (18)	0.0525 (13)	0.0637 (16)	−0.0034 (12)	0.0059 (14)	0.0114 (11)
C13	0.0781 (16)	0.0486 (12)	0.0551 (14)	−0.0018 (11)	0.0107 (12)	−0.0078 (11)

### Geometric parameters (Å, º)

**Table:**

N1—C7	1.327 (2)	C6—C7	1.490 (3)
N1—O1	1.422 (2)	C7—C8	1.425 (3)
O1—C9	1.364 (2)	C8—C9	1.351 (3)
O2—C10	1.244 (2)	C8—H8	0.9300
C1—C2	1.396 (3)	C9—C10	1.506 (3)
C1—C6	1.401 (3)	C10—N2	1.349 (2)
C1—H1	0.9300	N2—C12	1.469 (2)
C2—C3	1.384 (3)	N2—C13	1.471 (2)
C2—H2	0.9300	C12—H12A	0.9600
C3—C4	1.380 (3)	C12—H12B	0.9600
C3—H3	0.9300	C12—H12C	0.9600
C4—C5	1.393 (3)	C13—H13A	0.9600
C4—H4	0.9300	C13—H13B	0.9600
C5—C6	1.399 (3)	C13—H13C	0.9600
C5—H5	0.9300		
			
C7—N1—O1	105.64 (16)	C9—C8—H8	127.4
C9—O1—N1	108.28 (14)	C7—C8—H8	127.4
C2—C1—C6	119.9 (2)	C8—C9—O1	109.81 (16)
C2—C1—H1	120.0	C8—C9—C10	128.71 (18)
C6—C1—H1	120.0	O1—C9—C10	121.42 (16)
C3—C2—C1	120.7 (2)	O2—C10—N2	123.01 (18)
C3—C2—H2	119.6	O2—C10—C9	116.34 (17)
C1—C2—H2	119.6	N2—C10—C9	120.64 (17)
C4—C3—C2	119.4 (2)	C10—N2—C12	118.31 (17)
C4—C3—H3	120.3	C10—N2—C13	126.38 (16)
C2—C3—H3	120.3	C12—N2—C13	115.05 (16)
C3—C4—C5	120.8 (2)	N2—C12—H12A	109.5
C3—C4—H4	119.6	N2—C12—H12B	109.5
C5—C4—H4	119.6	H12A—C12—H12B	109.5
C4—C5—C6	120.2 (2)	N2—C12—H12C	109.5
C4—C5—H5	119.9	H12A—C12—H12C	109.5
C6—C5—H5	119.9	H12B—C12—H12C	109.5
C5—C6—C1	118.91 (18)	N2—C13—H13A	109.5
C5—C6—C7	119.67 (18)	N2—C13—H13B	109.5
C1—C6—C7	121.42 (18)	H13A—C13—H13B	109.5
N1—C7—C8	110.98 (17)	N2—C13—H13C	109.5
N1—C7—C6	119.88 (17)	H13A—C13—H13C	109.5
C8—C7—C6	129.14 (17)	H13B—C13—H13C	109.5
C9—C8—C7	105.29 (17)		
			
C7—N1—O1—C9	−0.6 (2)	N1—C7—C8—C9	−1.2 (2)
C6—C1—C2—C3	−1.1 (3)	C6—C7—C8—C9	179.07 (19)
C1—C2—C3—C4	0.7 (4)	C7—C8—C9—O1	0.8 (2)
C2—C3—C4—C5	−0.1 (4)	C7—C8—C9—C10	177.71 (19)
C3—C4—C5—C6	−0.1 (3)	N1—O1—C9—C8	−0.1 (2)
C4—C5—C6—C1	−0.2 (3)	N1—O1—C9—C10	−177.31 (16)
C4—C5—C6—C7	179.77 (19)	C8—C9—C10—O2	−24.5 (3)
C2—C1—C6—C5	0.8 (3)	O1—C9—C10—O2	152.16 (19)
C2—C1—C6—C7	−179.19 (19)	C8—C9—C10—N2	155.4 (2)
O1—N1—C7—C8	1.1 (2)	O1—C9—C10—N2	−27.9 (3)
O1—N1—C7—C6	−179.12 (15)	O2—C10—N2—C12	−2.0 (3)
C5—C6—C7—N1	−165.58 (19)	C9—C10—N2—C12	178.11 (18)
C1—C6—C7—N1	14.4 (3)	O2—C10—N2—C13	171.7 (2)
C5—C6—C7—C8	14.1 (3)	C9—C10—N2—C13	−8.2 (3)
C1—C6—C7—C8	−165.9 (2)		

### Hydrogen-bond geometry (Å, º)

**Table:**

*D*—H···*A*	*D*—H	H···*A*	*D*···*A*	*D*—H···*A*
C8—H8···O2^i^	0.93	2.43	3.340 (3)	165

Symmetry code: (i) −*x*, −*y*, −*z*.
